# Paired box 5 is a frequently methylated lung cancer tumour suppressor gene interfering β‐catenin signalling and GADD45G expression

**DOI:** 10.1111/jcmm.12768

**Published:** 2016-02-04

**Authors:** Lijuan Zhao, Shuman Li, Lin Gan, Chunhong Li, Zhu Qiu, Yixiao Feng, Jisheng Li, Lili Li, Chen Li, Weiyan Peng, Can Xu, Zhenyu Wang, Tianli Hui, Guosheng Ren, Qian Tao, Tingxiu Xiang

**Affiliations:** ^1^ Chongqing Key Laboratory of Molecular Oncology and EpigeneticsThe First Affiliated Hospital of Chongqing Medical UniversityChongqingChina; ^2^ Department of ChemotherapyCancer CenterQilu HospitalShandong UniversityJinanShandongChina; ^3^ Cancer Epigenetics LaboratoryDepartment of Clinical OncologySir YK Pao Center for Cancer and Li Ka Shing Institute of Health SciencesThe Chinese University of Hong Kong and CUHK Shenzhen Research InstituteHong KongChina

**Keywords:** tumour suppressor, PAX5, methylation, cancer, β‐catenin, GADD45G

## Abstract

Recent studies suggest that paired box 5 (PAX5) is down‐regulated in multiple tumours through its promoter methylation. However, the role of PAX5 in non‐small cell lung cancer (NSCLC) pathogenesis remains unclear. The aim of this study is to examine PAX5 expression, its methylation status, biological functions and related molecular mechanism in NSCLC. We found that PAX5 was widely expressed in normal adult tissues but silenced or down‐regulated in 88% (7/8) of NSCLC cell lines. PAX5 expression level was significantly lower in NSCLC than that in adjacent non‐cancerous tissues (*P* = 0.0201). PAX5 down‐regulation was closely associated with its promoter hypermethylation status and PAX5 expression could be restored by demethylation treatment. Frequent PAX5 promoter methylation in primary tumours (70%) was correlated with lung tumour histological types (*P* = 0.006). Ectopic expression of PAX5 in silenced lung cancer cell lines (A549 and H1975) inhibited their colony formation and cell viability, arrested cell cycle at G2 phase and suppressed cell migration/invasion as well as tumorigenicity in nude mice. Restoration of PAX5 expression resulted in the down‐regulation of β‐catenin and up‐regulation of tissue inhibitors of metalloproteinase 2, GADD45G in lung tumour cells. In summary, PAX5 was found to be an epigenetically inactivated tumour suppressor that inhibits NSCLC cell proliferation and metastasis, through down‐regulating the β‐catenin pathway and up‐regulating GADD45G expression.

## Introduction

Lung cancer is the leading cause of cancer‐related deaths worldwide. According to the prediction by American Cancer Society, lung cancer will have the highest incidence (221,200) and the second highest mortality (158,040) in the United States in 2015 1. Non‐small cell lung cancer (NSCLC) accounts for approximately 75–80% of all lung cancers 2. Adenocarcinoma is the most common subtype in NSCLC, which corresponds to about 40% of lung cancer 3. Although the mechanism leading to cancer development remains unclear, epigenetic inactivation of tumour suppressor genes (TSG) by promoter hypermethylation is increasingly recognized to play a crucial role in lung cancer.

Paired box 5 (PAX5) gene is located to chromosome 9p13 4, 5, encoding a nuclear transcription factor that is involved in control of organ development and tissue differentiation 6. PAX5 was originally identified as B‐cell‐specific activator protein that has an essential role in the early stages of B‐cell differentiation, neural development and spermatogenesis 7, 8, 9. PAX5 is widely expressed in normal adult and embryonic tissues 10, as well as in some tumour tissues including oral squamous‐cell carcinoma and leukoplakia 11, superficial bladder transitional cell carcinoma 12 and some neuroendocrine tumours such as SCLC, large cell neuroendocrine carcinoma, and pulmonary carcinoid 13. However, PAX5 expression is low or lost in most human cancers such as childhood ependymoma, NSCLC, mesothelioma, pancreatic, esophageal, head and neck cancer, lymphoma, and lymphocytic leukaemia 13, 14, 15, 16. Recent studies have shown that PAX5 is silenced by promoter hypermethylation in some types of human cancers including hepatocellular carcinoma 17, gastric cancer 10, breast cancer 18, lung cancer and lymphoid neoplasm 14, 19. Palmisano *et al*. showed such expression pattern in limited cases of breast cancer and lung cancer 14. These findings suggest that PAX5 may act as a putative TSG, but have yet to identify the function of PAX5 in NSCLC. In this study, we sought to investigate the epigenetic regulation of PAX5 in NSCLC by utilizing more number of tissue samples and more cell lines. And for the first time, we provided evidence of PAX5 acting as a tumour suppressor in NSCLC from biological function and molecular levels, thus laid the groundwork for potential clinical application.

## Materials and methods

### Tumour cell lines

Eight NSCLC cell lines (A427, A549, H1299, H1395, H1650, H1975, H292 and H358) were obtained from the Chinese University of Hong Kong, Hong Kong, China. They were cultured in RPMI 1640 medium (Gibco BRL, Rockville, MD, USA) supplemented with 10% foetal bovine serum (FBS; Gibco, Carlsbad, CA, USA) and 1% Penicillin Streptomycin (Gibco BRL) and incubated in 5% CO_2_ at 37°C.

### Patients and primary human samples

A total of 47 cases of primary NSCLC tissues, 40 cases of adjacent non‐tumour tissues and 16 cases of normal lung tissues were obtained from the department of thoracic surgery were obtained from the department of pneumology, the First Affiliated Hospital of Chongqing Medical University. All of the samples were verified by histology. The clinicopathological features, such as age, gender, smoking status, histological type, TNM stage, differentiated degree, lymph node metastasis and distant metastasis were also obtained. Informed consent was given to every patient. The study protocol was approved by the Clinical Research Ethics Committee of the Chongqing Medical University (Approval Notice 2010/2012(23).

### RNA extraction, semi‐quantitative PCR and real‐time PCR analyses

Human normal adult tissue RNA samples were purchased commercially (Stratagene, La Jolla, CA, USA or Millipore Chemicon, Billerica, MA, USA). Total RNA was extracted from 8 NSCLC cell lines and 23 paired tumour tissues and their corresponding adjacent tissues using Trizol reagent (Invitrogen, Carlsbad, CA, USA) and 1 μg of total RNA was reverse transcribed into 20 μl cDNA using a Reverse Transcription System (Promega, Madison, WI, USA). For semi‐quantitative PCR, the PAX5 gene was amplified using Go‐Taq DNA polymerase (Promega) under reaction conditions of 95°C for 30 sec., 55°C for 30 sec. and 72°C for 30 sec. over 32 cycles, with β‐actin as internal control. Real‐time PCR was performed with ABI SYBR green on an ABI 7500 real‐time PCR detection system (Applied Biosystems, California, USA) according to the manufacturer's instructions. GAPDH was used as a control. Each sample was tested in triplicate. Relative target gene expression was calculated by the 2^−ΔΔCt^ method. All primers are shown in Table 1.

**Table 1:** List of primers used in this study

PCR	Primer	Sequence (5′–3′)	Product size (bp)	PCR cycles	Annealing temperature (°C)
MSP	*PAX5* m1	AAATAAAAATTCGGTTTGCGTTC	105	41	60
	*PAX5* m2	AAACATACGCTTAAAAATCGCG			
	*PAX5*u1	TAAAAATAAAAATTTGGTTTGTGTTT	111	41	58
	*PAX5*u2	TTAAAACATACACTTAAAAATCACA			
RT‐PCR	*PAX5*‐F	GTCCATTCCATCAAGTCCTG	230	32	55
	*PAX5*‐R	TTGCTGACACAACCATGGCT			
	β‐actin‐F	TCCTGTGGCATCCACGAAACT	315	23	55
	β‐actin‐R	GAAGCATTTGCGGTGGACGAT			
	p53‐F	AAGACTCCAGTGGTAACCTACTG	632	32	55
	p53‐R	ATCTAAGCTGGTATGTCCTACTC			
	GADD45G‐F	AACTAGCTGCTGGTTGATCG	178	32	55
	GADD45G‐R	CGTTCAAGACTTTGGCTGAC			
	CDC2‐F	CGCAACAGGGAAGAACAG	135	32	55
	CDC2‐R	CGAAAGCCAAGATAAGCAAC			
	TIMP2‐F	AAGATGCACATCACCCTCTG	176	32	55
	TIMP2‐R	GTGACCCAGTCCATCCAGA			
	MMP2‐F	TTTGACGGTAAGGACGGACTC	346	32	55
	MMP2‐R	CCTGGAAGCGGAATGGAA			
	MMP9‐F	TCTATGGTCCTCGCCCTGAA	219	32	55
	MMP9‐R	CATCGTCCACCGGACTCAAA			
qRT‐PCR	*PAX5*‐F	TGGCAGGTATTATGAGACAGG	129	40	60
	*PAX5*‐R	CAGGCAAACATGGTGGGATT			
	GAPDH‐F	CCAGCAAGAGCACAAGAGGAA	114	40	60
	GAPDH‐R	CAAGGGGTCTACATGGCAACT			

### DNA extraction and methylation‐specific PCR

Genomic DNA was extracted from the NSCLC tissues or cell lines using a QIAamp DNA mini kit (Qiagen, Hilden, Germany). One microgram of genomic DNA was modified by sodium bisulphite using the EZ DNA methylation‐gold kit (ZYMO Research, Irvine, CA, USA). The bisulfite‐modified DNA was then amplified using primers that specifically amplify either methylated or unmethylated sequences of the PAX5 gene (Table 1). Amplification was carried out for 40 cycles (30 sec. at 94°C, 30 sec. at the annealing temperature, 30 sec. at 72°C), followed by 5 min. at 72°C.

### 5‐Aza and trichostatin A treatment

A549, H1395 and H1975 cells were treated with the DNA demethylating agent 5‐Aza (Sigma‐Aldrich, St. Louis, MO, USA) at 10 mM for 3 days, followed by the histone deacetylase inhibitor trichostatin A (TSA; Cayman Chemical Co, Ann Arbor, MI, USA) at 100 nM for another 24 hrs. 5‐Aza was replenished every day. Cells were then harvested for RNA extractions.

### Construction of PAX5‐expressing vectors

The full‐length open reading frame of PAX5 (NM_002448) was cloned into pReceiver‐M29 (GeneCopoeia, Rockville, MD, USA) with XmnI and XhoI to generate pNEG‐PAX5, which carry an EGFP tag in the N‐terminal of PAX5. Construction of recombinant pNEG‐PAX5 were validated by PCR and sequencing.

### Colony formation assay

A549 and H1975 cells were transfected with NEG‐PAX5 or NEG empty vector using Lipofectamine 2000 (Invitrogen) after reaching 70% confluence in six‐well plates. After 48 hrs, cells were selected with G418 (600 μg/ml for A549 cells and 400 μg/ml for H1975 cells; Invitrogen) for 2 weeks to obtain stably transfected cell lines which were verified by RT‐PCR and western blotting. A549 and H1975 cells stably expressing PAX5 or NEG were counted and each kind of cells plated 200, 400, 800 cells/well into six‐well plates, respectively, then cultured in selection medium containing G418 for another 2 weeks. Colonies with cell numbers greater than 50 cells per colony were fixed in 4% paraformaldehyde and stained with 0.1% crystal violet solution. All the experiments were performed in triplicate in three independent experiments.

### CCK8 viability assay

Cell viability was assessed using Cell Counting Kit‐8 (CCK8) (Dojindo Laboratories, Kumamoto, Japan). A549 and H1975 cells stably transfected with PAX5‐expressing or NEG empty vector were seeded into four 96‐well plates at a density of 2 × 10^3^ cells/well with 100 μl of complete culture medium. After cells adhered, the supernatant was removed and 100 μl of serum‐free medium containing 10 μl of CCK8 was added to each well and incubated at 37°C for 2 hrs. Absorbance was measured at 450 nm using a microplate reader. The remaining three plates were tested after incubation for 24, 48 and 72 hrs. All experiments were repeated independently for three times.

### Cell cycle

The stably transfected A549 and H1975 cells with NEG‐PAX5 or empty vector were collected and washed twice in PBS, then fixed for 24 hrs in ice‐cold 70% ethanol and treated with 50 μg/ml RNase‐A for 30 min. at 37°C. After staining with 50 μg/ml propidium iodide (BD Pharmingen, San Jose, CA, USA), the cells were sorted using a FACSCalibur machine (BD Biosciences, Franklin Lakes, NJ, USA) and cell‐cycle profiles were analysed by ModFit 3.0 software (Verity Software House, Topsham, ME, USA).

### Cell migration and invasion assay

Cell migration was assessed by scratch wound assay. Stable NEG‐PAX5‐ or empty vector‐ transfected A549 and H1975 cells were cultured in six‐well plates (5 × 10^5^ cells/well). When the cells reached 90% confluence, three scratch wounds across each well were created using a sterile P‐20 pipette tip. Cells were then washed twice with PBS to remove cell debris. The cells were then cultured with growth medium with 5% FBS to minimize cell proliferation. Images of the wound closure areas were taken at 0, 24, 48 and 72 hrs.

Cell migration was also assessed using Transwell chambers (8‐μm pore size; Corning, NY, USA). A549 and H1975 cells stably expressing NEG‐PAX5 or empty vector were collected and washed twice in serum‐free medium. Aliquots of 4 × 10^4^ cells/well were resuspended in 100 μl serum‐free medium and plated onto uncoated 8‐μm transwell filter inserts in 24‐well plates. The lower chambers contained 800 μl medium containing 10% FBS as a chemoattractant. The cells were incubated at 37°C in a 5% CO_2_ incubator for 24 hrs. Cells on the lower side of the inserts were fixed in 4% paraformaldehyde for 30 min. and stained with 0.1% crystal violet for 15 min. Non‐migratory cells in the upper chamber were removed with a cotton swab. Cells were photographed and counted under a microscope in seven random fields.

To evaluate their invasion ability, cells were allowed to invade Matrigel^™^‐coated transwell filters (Matrigel^™^: serum‐free medium = 1:5, 100 μl/chamber). 2 × 10^4^ cells/well were resuspended in 100 μl serum‐free medium and plated onto Matrigel^™^‐coated transwell filters then incubated for 24 hrs. At the end of the culture period, invaded cells on the bottom of the membrane were fixed, stained and counted. All experiments were independently repeated three times.

### *In vivo* tumorigenicity

NEG‐PAX5 or empty vector stably transfected H1975 cells (5 × 10^6^ cells suspended in 150 μl serum‐free medium) were injected subcutaneously into the dorsal flank of 4‐week‐old female BALB/c nude mice (6/group), separately. After 1 week, tumours were clearly visible, and tumour length and width were measured every 3 days for 19 days using microcalipers. Tumour volume (mm^3^) was calculated using the following equation: volume = 0.5 × length × width^2^. When the tumour length reached 1 cm, the mice were killed and the tumours were removed. All tumours were weighed before fixation in 4% paraformaldehyde. Paraffin sections were prepared for immunohistochemical analyses. All experimental procedures were approved by the Animal Ethics Committee of the Experimental Animal Center of the Chongqing Medical University, Chongqing, China.

### Western blot analysis

A549 and H1975 cells were harvested and total protein was extracted at 48 hrs after transfection with NEG‐PAX5 or NEG empty vector. Protein concentration was measured by the bicinchoninic acid assay. The protein samples mixed with loading buffer were denatured by heating at 100°C for 10 min. Forty micrograms of protein from each sample were separated by 10–15% SDS‐PAGE gels (Invitrogen). After electrophoresis, the separated proteins were transferred onto polyvinylidene fluoride membranes (Millipore) and blocked with 5% bovine serum albumin for 1 hr. The membranes were then incubated overnight at 4°C with the following primary antibodies: PAX5 (1:5000, #3852‐1; Abcam, Cambridge, UK), β‐catenin (1:1000, #2677; Cell Signaling), active β‐catenin (1:1000, #4270; Cell Signaling, Danvers, MA, USA), cyclinD1 (1:1000, #2261; Epitomics), c‐Myc (1:10,000, #1472‐1; Epitomics, Burlingame, CA, USA), matrix metalloproteinase 2 (MMP2, 1:1000, ab86607; Abcam), MMP3 (1:1000, ab52915; Abcam), MMP7 (1:1000, #3801; Cell Signaling), MMP9 (1:5000, ab76003; Abcam), GADD45G (1:2000, ab140378; Abcam), tissue inhibitors of metalloproteinase 2 (TIMP2, 1:1000, ab1828; Abcam)and GAPDH (1:2000, #2261; Epitomics) was used as control. Next day the membranes were washed and incubated with a secondary goat anti‐rabbit IgG or goat anti‐mouse IgG monoclonal antibody conjugated with horseradish peroxidase at 1:1000 dilution for 1 hr at room temperature. The bands were detected with the enhanced chemiluminescence system. Proteins were visualized using ECL Plus Western Blotting Detection Reagents (RPN2132; GE HealthcareLife Sciences, Amersham, UK). The band intensities were quantified using Image‐Pro Plus 6.0 and the Quantity One 1‐D Analysis Software (Bio‐Rad, Hercules, CA, USA).

### Immunohistochemical staining

Samples of transplanted tumour tissues from nude mice were fixed in 4% paraformaldehyde before embedded in paraffin. The tissue sections were cut at 4 μm and dewaxed for 2 hrs in a 60°C incubator, then rinsed in absolute xylene for 10 min. The sections were then rehydrated through graded alcohol. Antigen retrieval was performed by boiling the slides in the antigen retrieval buffer for 20 min., then allowing them to cool naturally. The sections were then incubated in 3% hydrogen peroxide for 15 min. to block endogenous peroxidase activity and washed 3 times with PBS, then blocked with 5% FBS‐PBS solution for 15 min. at room temperature. After that, the sections were incubated at 4°C overnight with rabbit monoclonal antibody (PAX5 1:150 dilution, Ki67 Ready‐to‐use). Next day the slides were washed 3 times with PBS and incubated with the secondary antibody for 15 min. at room temperature, followed by colour development with DAB. The cell nuclei were counterstained with haematoxylin. Images were captured under a microscope.

### Statistical analysis

All statistical analyses were performed with SPSS software (version 16.0, SPSS Inc., Chicago, IL, USA). The significance of differences between the various groups was determined using the two‐tailed Student's *t*‐test. Data are presented as means ± S.D. of at least three independent experiments. The chi‐squared test was used to compare methylation status and clinicopathological parameters. Fisher's exact tests were used when appropriate. Differences were considered statistically significant if *P*‐values were less than 0.05.

## Results

### PAX5 is widely expressed in normal adult tissues and suppressed in NSCLC tumour tissues and cell lines

**Figure 1:**
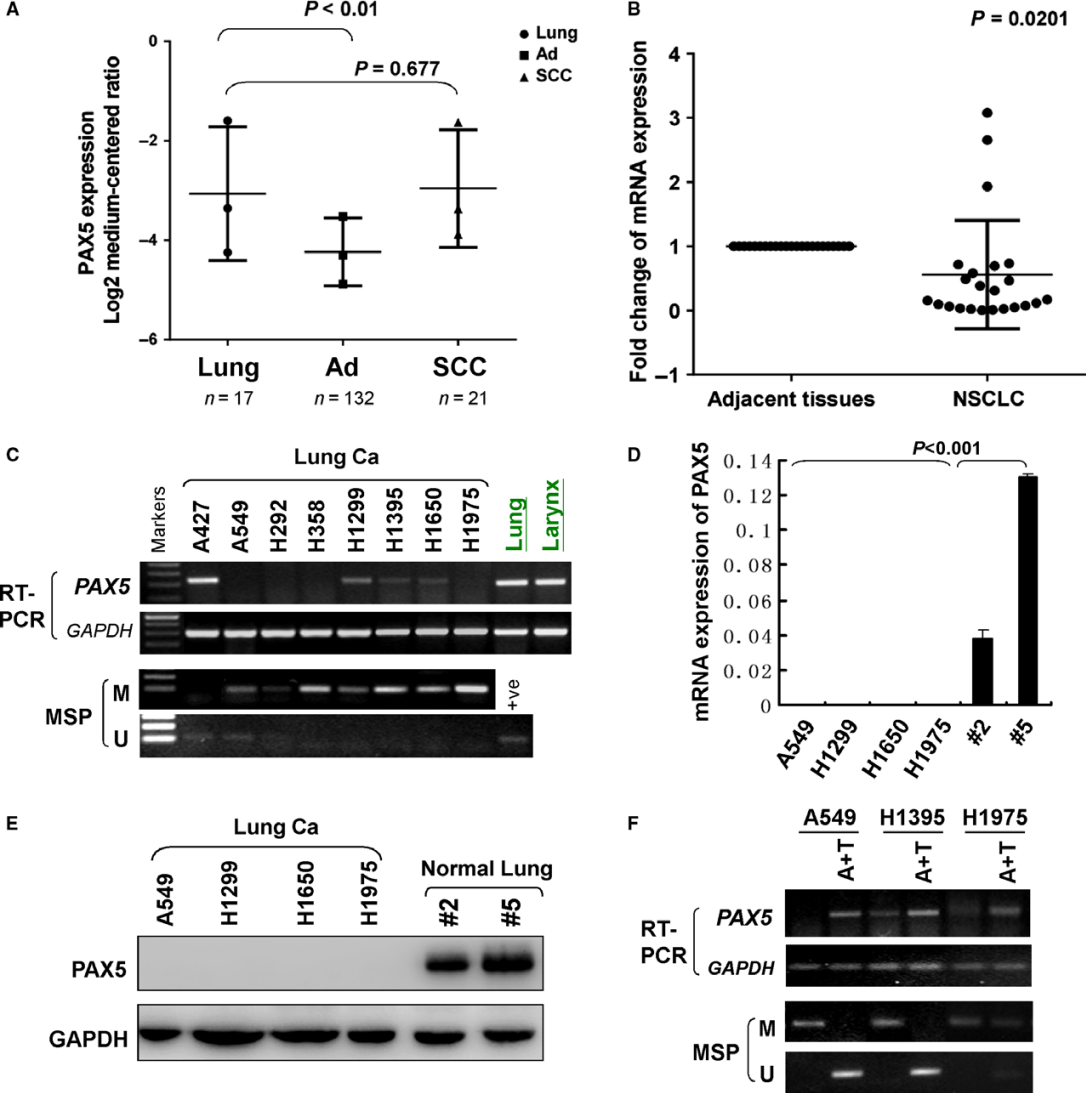
The expression and methylation status of PAX5 in NSCLC. (**A**) PAX5 expression (median of expression intensity) in NSCLC from Oncomine database (https://www.oncomine.org/). Ad: lung adenocarcinoma; SCC: squamous‐cell lung carcinoma. (**B**) PAX5 expression in primary NSCLC tissues and paired adjacent non‐tumour tissues was evaluated using real‐time PCR (*n* = 23). PAX5 expression level was normalized with the mRNA level. (**C**) PAX5 expression by semi‐quantitative PCR and methylation status of the PAX5 promoter by MSP in a panel of human NSCLC cell lines and the PAX5 expression of normal lung and larynx tissue. β‐actin was used as a control. +Ve, positive control; M: methylated; U: unmethylated. (**D**) PAX5 mRNA expression in four NSCLC cell lines and two normal lung tissue samples by qRT‐PCR. β‐actin was used as a control. (**E**) PAX5 protein expression in four NSCLC cell lines and two normal lung tissue samples by western blot. GAPDH was used as a control. (**F**) Pharmacological demethylation restores expression of PAX5. A549, H1395 and H1975 cells were treated with Aza combined with TSA (A+T). PAX5 expression by semi‐quantitative PCR and methylation status of the PAX5 promoter by MSP after A+T treatment. GAPDH was used as a control.

We used semi‐quantitative PCR to examine the messenger RNA (mRNA) expression of PAX5 in normal adult tissues. We found that PAX5 was highly expressed in oesophagus, stomach, colon, larynx, trachea, lung, prostate, brain, spleen, lymph node, and weakly expressed in pancreas, rectum, breast, cervix, ovary, kidney, testis, placenta, while it was not expressed in heart, liver (data not shown). In NSCLC, PAX5 was significantly down‐regulated in lung adenocarcinoma, but not in squamous‐cell lung carcinoma (*P* < 0.01, Fig. 1A), through analysing the online microarray database (www.oncomine.org, Compendia Bioscience, Ann Arbor, MI, USA). Moreover, real‐time PCR results showed that PAX5 expression was down‐regulated in 83% (19/23) of primary NSCLC tumour tissues compared with their corresponding adjacent tissues (*P* < 0.0001, Fig. 1B). PAX5 was silenced or down‐regulated in seven out of eight (88%) NSCLC cell lines: silenced in A549, H292, H358, H1975 and down‐regulated in H1299, H1395, H1650, as show in Figure 1C. Two of each cell lines were chosen to verify the expression of mRNA compared with normal lung tissue samples *via* real‐time PCR (Fig. 1D). Western blot confirms the expression pattern of PAX5 on a protein level (Fig. 1E).

### PAX5 promoter is frequently methylated in NSCLC

Aberrant promoter methylation leading to the loss of gene expression of TSGs in tumours has been detected in human cancers 20, 21. To investigate whether the loss of PAX5 expression in NSCLC involves promoter hypermethylation, we explored the methylation status of the PAX5 promoter by methylation‐specific PCR (MSP). Full or partial methylation was detected in four of eight (50%) NSCLC cell lines (H292, H358, H1299, H1975), which was associated with silenced or reduced PAX5 expression, while normal lung and larynx tissue express significant level of PAX5 mRNA (Fig. 1C). To confirm whether the promoter methylation resulted in the silence of PAX5 in NSCLC, three cell lines, A549, H1395 and H1975, which have silenced or reduced PAX5 expression, were treated with the DNA methyltransferase inhibitors (5‐aza‐2‐deoxycytidine, 5‐Aza) combined with the histone deacetylase inhibitor (TSA). As shown in Figure 1F, this treatment resulted in the restoration or up‐regulation of PAX5 expression and a change in the promoter methylation status, confirming that the silencing of PAX5 was mediated by promoter hypermethylation.

**Figure 2:**
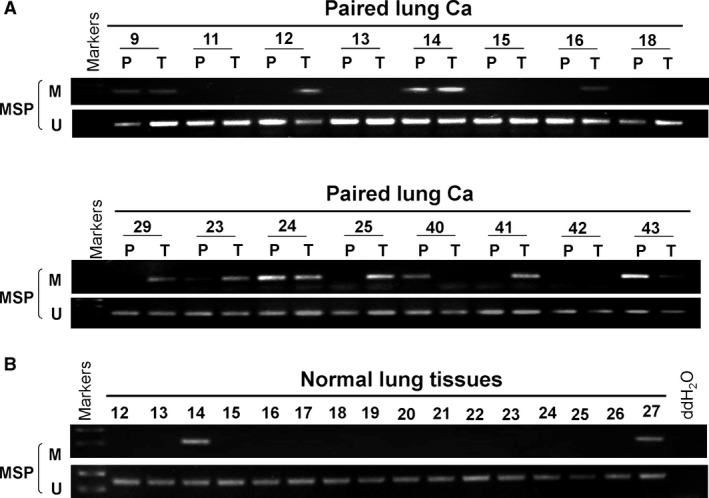
The methylation status of PAX5 in NSCLC by MSP. (**A**) Representative images of methylation of the PAX5 promoter in paired lung carcinoma tissues. P: paired adjacent non‐tumour tissues; T: tumour tissues. (**B**) Methylation of PAX5 in normal lung tissues. M: methylated; U: unmethylated.

In primary NSCLC tissues, adjacent non‐tumour tissues and normal lung tissues, the methylation frequencies of PAX5 promoter were 70% (33/47), 73% (29/40) and 13% (2/16), respectively (Table 2). The results of chi‐squared test showed that PAX5 methylation was associated with histological type (*P* = 0.006), among 33 cases of methylation tissues, 18 cases were adenocarcinoma (55%), 10 cases were squamous‐cell carcinoma (30%), 4 cases were lung adenosquamous carcinoma (12%), 1 case was large cell carcinoma (3%). No association was found between PAX5 methylation and age, gender, smoking status, TNM stage, differentiated degree and metastasis (*P* > 0.05, Table 3, Fig. 2A–C).

**Table 2:** Methylation status of the PAX5 promoter in primary Lung tumours

Samples	*PAX5* promoter		Frequency of methylation
	Methylation	Unmethylation	
NSCLC‐T (*n* = 47)	33	14	70%
NSCLC‐P (*n* = 40)	29	11	73%
NLT (*n* = 16)	2	14	13%

NSCLC‐T: non‐small cell lung cancer tissues; NSCLC‐P: paired non‐small cell lung cancer adjacent tissues; NLT: normal lung tissues.

**Table 3:** Clinicopathological features of PAX5 methylation in non‐small cell lung cancer

Clinicopathological features	Number (*n* = 47)	PAX5 methylation status		*P*‐value
		Methylated	Unmethylated	
Age (year)				
>60	28	21	7	0.518
≤60	19	12	7	
Gender				
Male	31	21	10	0.742
Female	16	12	4	
Smoking status				
Yes	28	18	10	0.344
No	19	15	4	
Histology				
Squamous carcinoma	22	10	12	0.006
Adenocarcinoma	19	18	1	
Lung adenosquamous carcinoma	5	4	1	
Large cell carcinoma	1	1	0	
Differentiation				
Moderately/Well	19	10	9	0.055
Poorly	10	7	3	
Unknown	18	16	2	
TNM stage				
I	22	13	9	0.395
II	9	8	1	
III	12	9	3	
IV	4	3	1	
Lymph node metastasis				
Yes	21	16	5	0.528
No	26	17	9	
Distant metastasis				
Yes	4	3	1	1
No	43	30	13	

### PAX5 inhibits NSCLC cell proliferation

**Figure 3:**
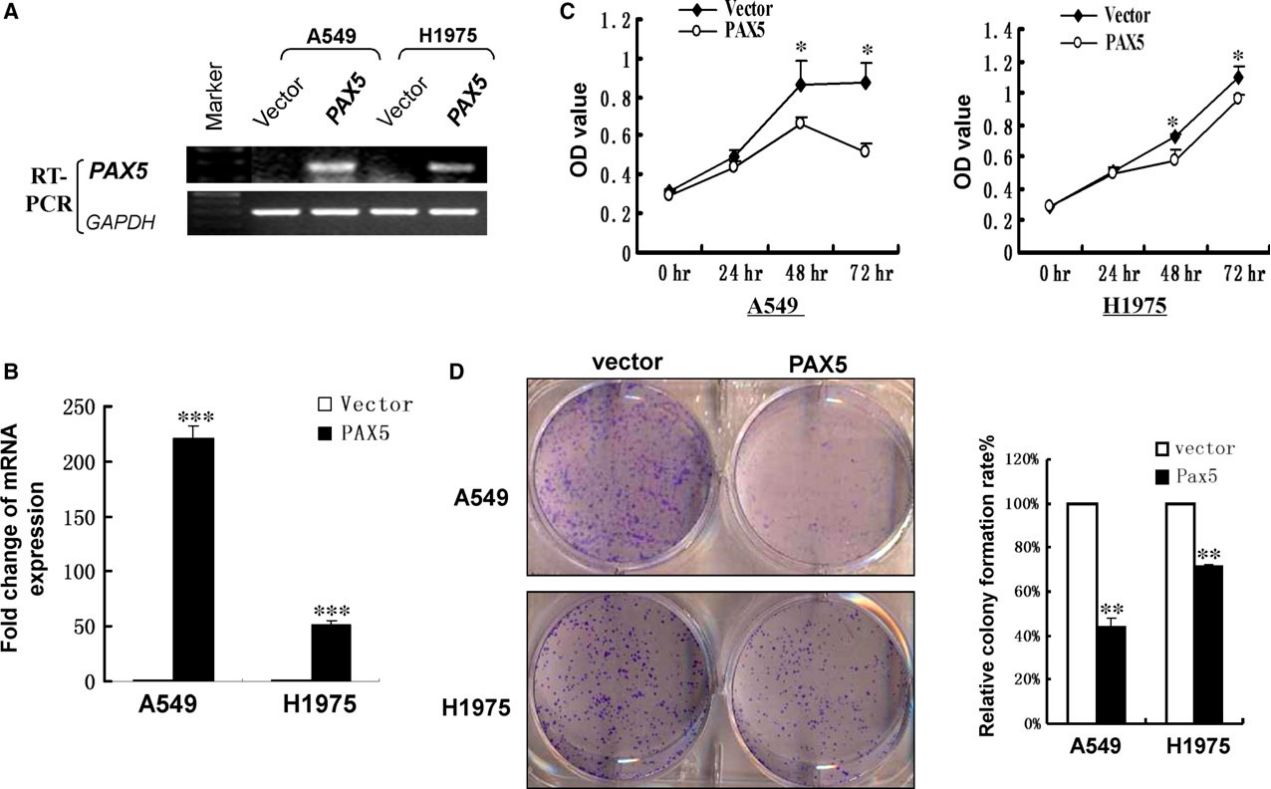
Ectopic expression of PAX5 inhibited cell proliferation in A549 and H1975 cell lines. Re‐expression of PAX5 in A549 and H1975 cell lines was confirmed by semi‐quantitative PCR (**A**) and real‐time PCR (**B**). (**C**) CCK‐8 proliferation assay for A549 and H1975 cells transfected with PAX5 compared with control at each time‐point, *P < 0.05. (**D**) Representative colony formation assay (left) and quantitative analysis of colony formation (right). The number of G418‐resistant colonies in vector‐transfected cells was set as 100%. Values are expressed as mean ± S.D. for three experiments (**P < 0.01, *** p < 0.001).

The observation that PAX5 was frequently silenced by promoter hypermethylation in NSCLC but not in normal lung tissues led us to hypothesize that PAX5 is a candidate tumour suppressor in NSCLC. We therefore examined the growth‐inhibiting effect through re‐expression of PAX5 in A549 and H1975 cells, which had no intrinsic PAX5 expression. Stable transfection was performed in two lung adenocarcinoma cell lines A549 and H1975 that had no intrinsic PAX5 expression and re‐expression of PAX5 was confirmed by semi‐quantitative PCR and real‐time PCR (Fig. 3A and B). As shown in Figure 3C, the absorbance at 450 nm in PAX5‐overexpressed cells decreased significantly after 2 days in a CCK8 cell proliferation assay, compared to cells transfected with the empty vector. The suppressive effect on cancer cell growth was further confirmed by colony formation assay. As shown in Figure 3D, the colony number in cells transfected with PAX5 was significantly lower than that of those transfected with empty vector in A549 and H1975 cells. The ability to suppress proliferation implies that PAX5 is a potential tumour suppressor in NSCLC.

### PAX5 induces G2 phase cell cycle arrests in NSCLC cells

**Figure 4:**
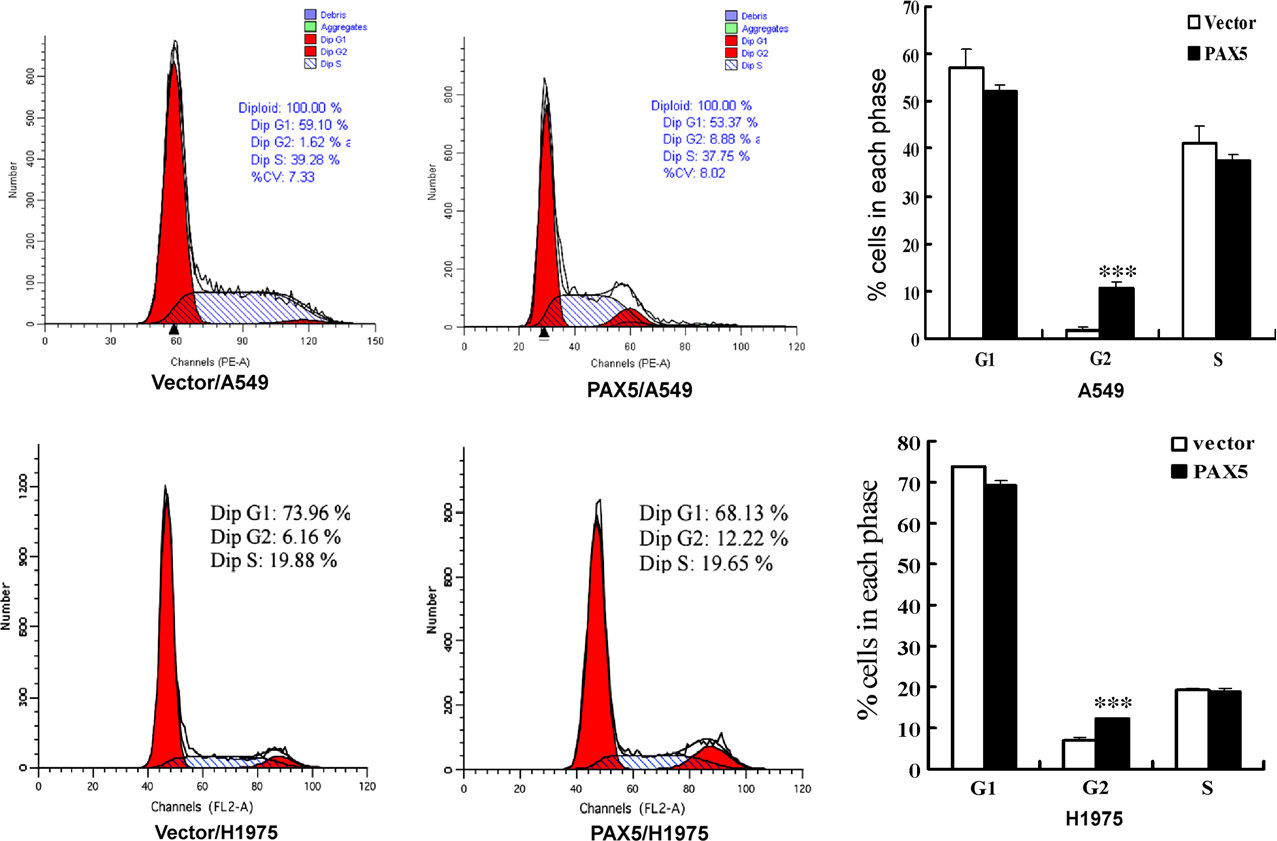
Ectopic expression of PAX5 induced cell cycle arrest at G2 phase. The cell cycle distribution of vector‐, PAX5‐transfected A549 and H1975 cells was detected by flow cytometry analysis. The distribution and percentage of cells in G1, S and G2 phase of the cell cycle are indicated, ***P < 0.001.

We next investigated the effect of PAX5 on cell cycle distribution. FACS analysis of PAX5‐transfected A549 and H1975 cells revealed a significant increase in the number of cells accumulating in the G2 phase (*P* < 0.001), accompanied by a significant decrease in the number of cells in the G1 and S phases compared with controls, confirming the inhibitory effect of PAX5 on cell proliferation (Fig. 4).

### PAX5 inhibits NSCLC cell migration and invasion

**Figure 5:**
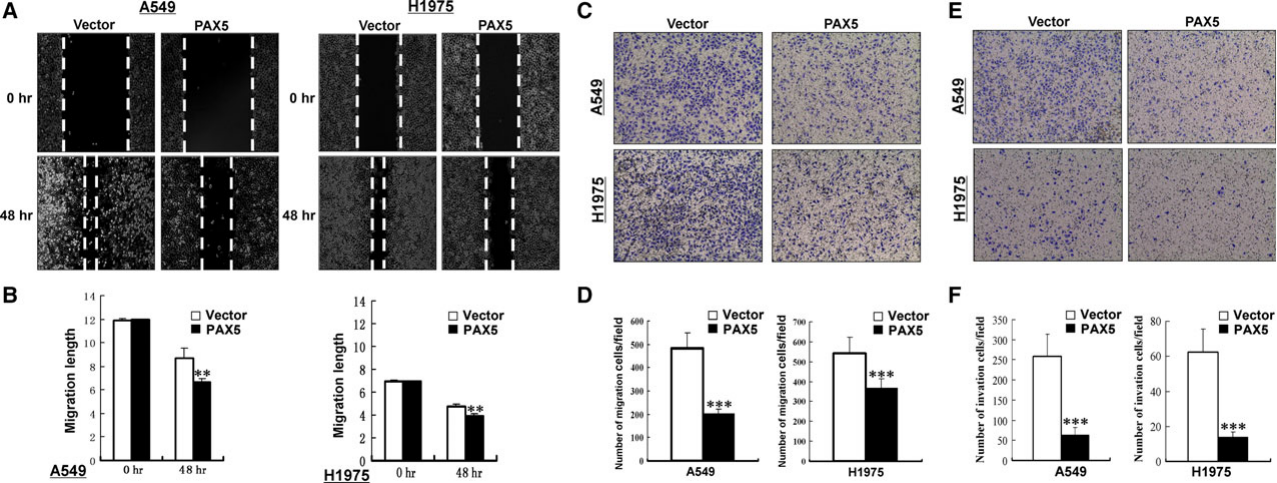
Ectopic expression of PAX5 inhibited migration and invasion in A549 and H1975 cells. (**A**) Wound healing assay. Photographs were taken at 0 and 48 hrs to determine the different mobility between Vector‐ and PAX5‐transfected A549 and H1975 cells. (**B**) Migration length of Vector‐ and PAX5‐transfected A549 and H1975 cells was shown as mean ± S.D., **P < 0.01. Representative images of the transwell migration and invasion assay (**C** and **E**). The pictures were taken at 24 hrs after seeding (magnification, ×200). The numbers of migrated and invaded cells were counted in seven representative high power fields per transwell (**D** and **F**). The values are shown as mean ± S.D. Three independent experiments were carried out in triplicate, ***P < 0.001.

To investigate the effect of PAX5 on NSCLC cell migration, a monolayer wound‐healing assay was performed. The results shown in Figure 5A and B demonstrate that ectopic expression of PAX5 in A549 and H1975 cells significantly inhibited the closure of wound gaps, compared to the control groups (*P* < 0.01). For the quantitative assessment of cell metastasis and invasiveness, we performed a transwell migration and invasion assay. The results showed that the number of migrated and invaded cells was significantly reduced in cells with ectopic expression of PAX5 than the control cells (*P* < 0.001, Fig. 5C–F), suggesting that PAX5 inhibits the migration and invasion of NSCLC cells.

### PAX5 inhibits xenografted tumour growth in nude mice

**Figure 6:**
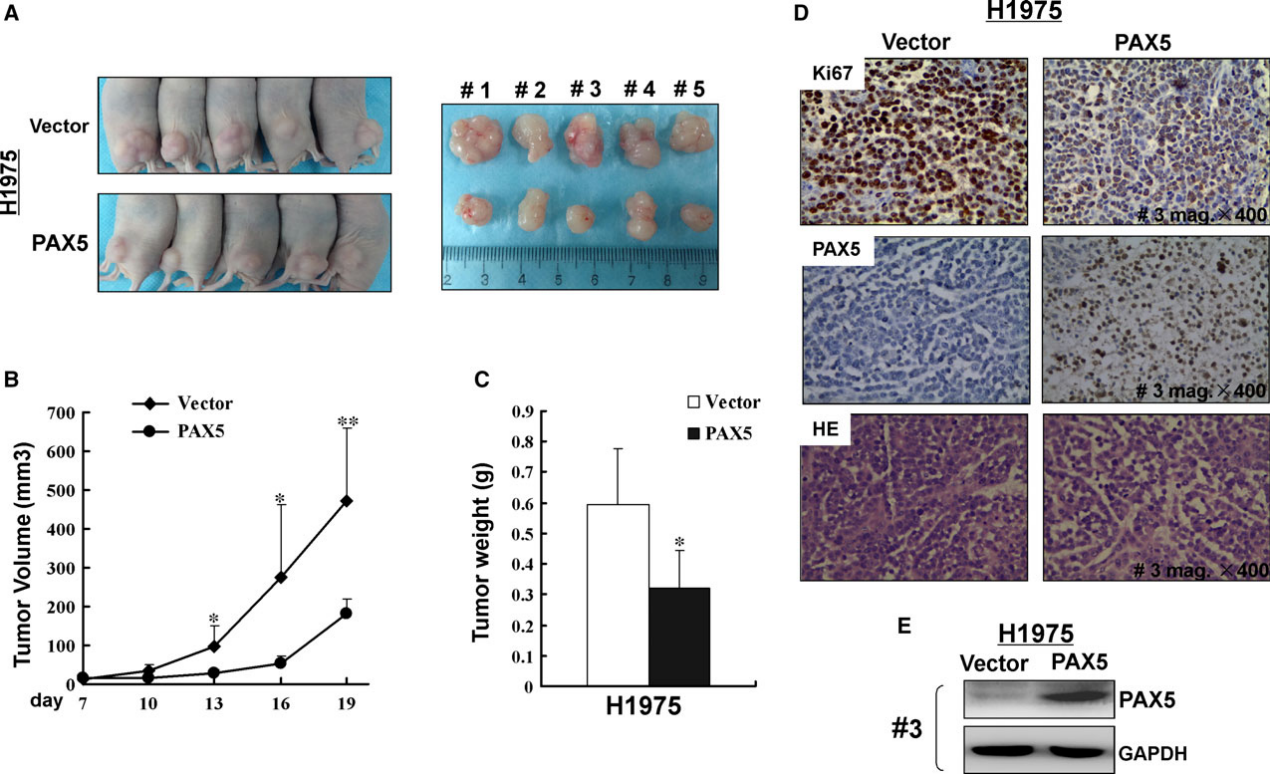
PAX5 inhibited the tumorigenicity of NSCLC *in vivo*. (**A**) Representative images of tumour growth in nude mice. (**B**) Growth curve for the PAX5‐expressing tumour compared to the control in nude mice. *P < 0.05, **P < 0.01. (**C**) Tumour weigh of PAX5‐expressing cells in nude mice compared with control tumours, *P < 0.05. (**D**) Representative photographs of HE staining and IHC analyses of the expression of PAX5 and Ki67 in tumours from nude mice (magnification, ×400). (**E**) Protein expression of PAX5 in both neoplasms from mouse #3. GAPDH was used as a control.

We further tested whether PAX5 is able to suppress the growth of NSCLC cells *in vivo*. H1975 cells stably transfected with PAX5 or empty vector formed tumours in nude mice (Fig. 6A). The subcutaneous tumour growth curve is shown in Figure 6B. The tumour volume of tumours derived from PAX5‐transfected was significantly smaller than that of controls (*P* < 0.01). At the end of the experiment, tumours were isolated and weighed. The mean weight of tumours formed by PAX5‐transfected cells was significantly lower than that of vector control formed tumours (Fig. 6C, *P* < 0.05), indicating that PAX5 can inhibit NSCLC cell grow tumours *in vivo*.

Expression of PAX5 protein in isolated tumour tissues was confirmed by immunohistochemical staining of PAX5 monoclonal antibody in the nucleus and by western blot (Fig. 6D and E). Additionally, the Ki‐67 stain that reflects cell proliferation was reversely correlated to the expression of PAX5 (Fig. 6D).

### The tumour suppressive property of PAX5 is mediated by β‐catenin signalling pathways

**Figure 7:**
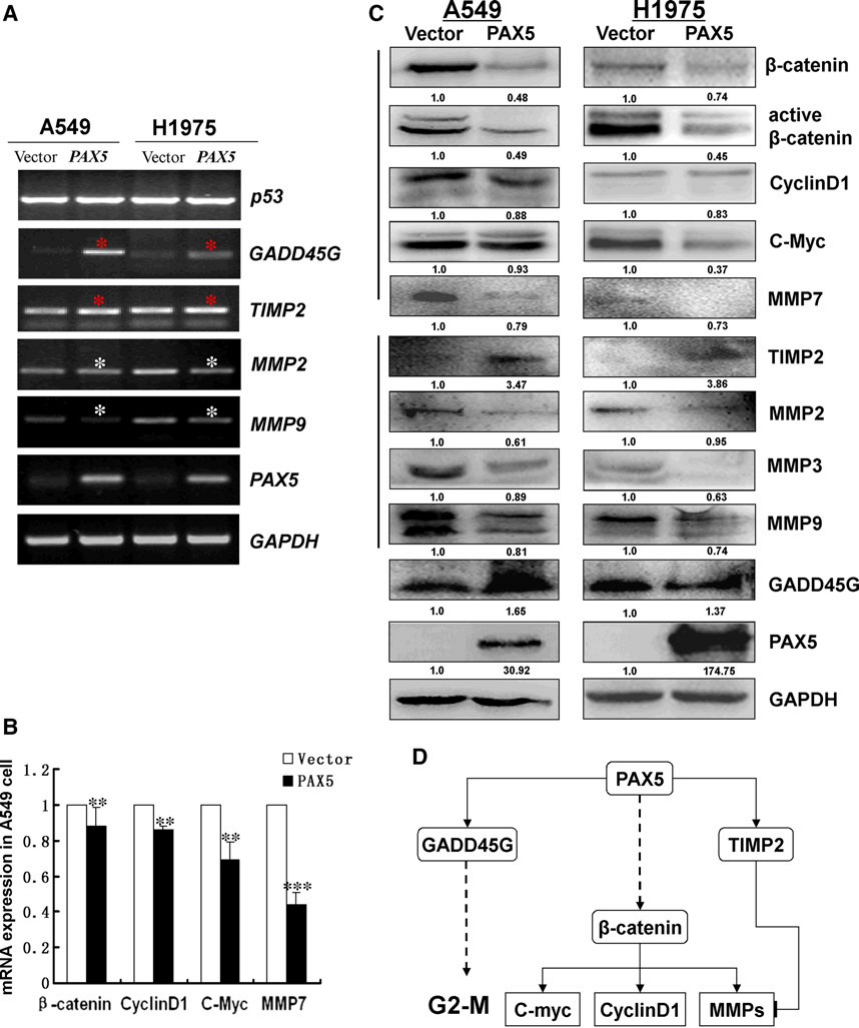
(**A**) Expression of PAX5, p53, GADD45G, CDC2, TIMP2, MMP2 and MMP9 was evaluated by semi‐quantitative PCR in vector‐ and PAX5‐transfected A549 and H1975 cells. GAPDH was used as an internal control, *P < 0.05. (**B**) The mRNA expression of β‐catenin, cyclin D1, C‐Myc and MMP7 in vector‐ and PAX5‐transfected A549 cells by qRT‐PCR. β‐actin was used as a control. **P < 0.01, ***P < 0.001. (**C**) Effects of ectopic expression of PAX5 on protein expression of β‐catenin, active β‐catenin, cyclin D1, C‐Myc and GADD45G using western blot. GAPDH was used as an internal control. (**D**) Schematic diagram for the mechanisms of anti‐tumorigenesis functions of PAX5 deriving from semi‐quantitative PCR and western blot.

To elucidate the downstream signalling pathways mediated by PAX5 in tumour inhibition, we performed semi‐quantitative PCR and Western blot to evaluate the expression of components of the β‐catenin signalling pathways. In A549 and H1975 cell lines, PAX5 decreased the expression of β‐catenin and its downstream target genes, c‐Myc, cyclin D1 and MMP7. At the same time, PAX5 up‐regulated TIMP2 which can inhibit MMPs, including MMP2, MMP3 and MMP9. PAX5 also enhanced GADD45G and suppressed CDC2 to arrest cells at G2 phase (Fig. 7A–C).

## Discussion

Histologically, NSCLC consists of adenocarcinoma, squamous carcinoma, large cell carcinoma and other subtypes. Data from Oncomine database showed that PAX5 is down‐regulated significantly in lung adenocarcinoma but not squamous carcinoma. In this study we found that PAX5 was generally highly expressed in normal adult tissues while the expression of which was frequently down‐regulated or lost in NSCLC cell lines, and primary tumour tissues compared with their adjacent non‐tumour tissues. The reduced expression was attributed to hypermethylation of the PAX5 gene promoter as evaluated by MSP. In addition, we found that methylation of PAX5 promoter was correlated with lung adenocarcinoma. These results indicate that the reduced expression of PAX5 was most probably attributed to promoter hypermethylation. Our results were consistent with a previous report that PAX5 genes were methylated in lung tumours 14. Furthermore, silencing of PAX5 could be reversed by demethylation treatment with the methylation transferase inhibitor 5‐Aza and histone deacetylase inhibitor TSA, suggesting that epigenetic modification is an important regulatory mechanism of PAX5 expression and activity in NSCLC. The A549 and H1395 cell lines had no promoter methylation, but could be reversed by TSA, suggesting that the methylation of PAX5 promoter at a different place may not be covered by one pair of MSP primer and the histone medication also contributes to PAX5 gene silencing.

The biological functions of PAX5 in NSCLC were further investigated by re‐expression of PAX5 both *in vitro* and *in vivo* assays. Ectopic expression of PAX5 in the silenced A549 and H1975 cell lines showed a significant proliferation suppressing effect shown by cell proliferation, colony formation and cell cycle distribution assays. The diminution of tumour growth by PAX5 re‐expression was also detected by xenografted tumour formation in nude mice. In addition, PAX5 was able to arrest A549 and H1975 cells at G2 phase as well as inhibiting cell migration and invasion. These results strongly suggest that PAX5 functions as a tumour suppressor in NSCLC cells.

Wnt signalling pathways alterations are prominent in human malignancies. Wnt proteins binds to members of the Frizzled (FZD) family of receptors, forming a stable receptor complex to initiate Wnt/β‐catenin signalling cascade to regulate the expression of target genes. In this pathway, increased expression of c‐Myc and cyclin D1 can accelerate tumour formation, playing a central role in the deregulation of cell cycle 22. There is growing evidence that the Wnt pathway is important in NSCLC tumorigenesis and prognosis 23. In the present study, we found that ectopic expression of PAX5 inhibited β‐catenin signalling and its downstream targets c‐Myc and cyclin D1. However, the mechanism of PAX5 on Wnt pathway needs to be further studied.

We found that percentage of cells in the G2 phase increased from 6.9% in the control cells to 12.1% in the PAX5‐transfected A549 cells and from 1.6% to 10.5% in PAX5‐transfected H1975 cells. Several studies have suggested that the tumour suppressor p53 plays an important role in regulating the G2/M checkpoint. It was reported that PAX5 activates GADD45 expression through up‐regulating p53 17. GADD45G interact with both CDC2 and cyclinB1 to inhibit the kinase activity of the CDC2/cyclinB1 complex and regulate G2/M transition 24, 25, 26. Therefore, we hypothesize that the G2/M block and induction of apoptosis is driven by p53 induction. Indeed, we observed that PAX5 re‐expression caused cell cycle arrest at the G2/M checkpoint. However, we found that while re‐expression of PAX5 increased GADD45G expression and inhibited CDC2 expression, while it did not influence the expression of p53 in both A549 and H1975 cells, suggesting that an increase in GADD45G expression may not involve p53.

Tumour metastasis is the result of cancer cells invasion and the degradation of the extracellular matrix by specialized proteolytic enzymes. Among these enzymes, MMPs play important roles 27. Matrix metalloproteinases are highly expressed in various cancers, including colorectal cancer 28, prostate cancer and NSCLC^29^, ^30^, ^31^. Tissue inhibitors of metalloproteinases are specific inhibitors of MMPs, which inhibit the catalytic activity of MMPs through its amino‐terminal domain 32 Our results showed that re‐expression of PAX5 increased the TIMP2 mRNA level and inhibited the transcription of MMP2, MMP7, MMP9, suggesting that PAX5 can inhibit tumour invasion and metastasis through modulating these tissue remodelling enzymes.

## Conclusions

Our results strongly suggest PAX5 as a novel functional tumour suppressor that is inactivated by epigenetic regulation in NSCLC. PAX5 suppresses NSCLC by inhibiting cell proliferation and metastasis likely through interfering the β‐catenin signalling pathway and GADD45G expression.

## Conflicts of interest

The authors declare no conflict of interest.

## Author contribution

ZLJ, XC, FYX, LSM and LC performed experiments; QZ contributed samples; XTX and LL analysed data; RGS approved final version; XTX and QT conceived and supervised the study.
